# Melioidosis in the Lao People’s Democratic Republic

**DOI:** 10.3390/tropicalmed3010021

**Published:** 2018-02-19

**Authors:** David A.B. Dance, Manophab Luangraj, Sayaphet Rattanavong, Noikaseumsy Sithivong, Oulayphone Vongnalaysane, Manivanh Vongsouvath, Paul N. Newton

**Affiliations:** 1 Lao-Oxford-Mahosot Hospital-Wellcome Trust Research Unit, Microbiology Laboratory, Mahosot Hospital, Vientiane, Laos; manophab.l@tropmedres.ac (M.L.); Sayaphet@tropmedres.ac (S.R); Manivanh@tropmedres.ac (M.V.); Paul.Newton@tropmedres.ac (P.N.N.); 2 Centre for Tropical Medicine and Global Health, Nuffield Department of Clinical Medicine, Old Road Campus, University of Oxford, Oxford OX3 7FZ, UK; 3 Faculty of Infectious and Tropical Diseases, London School of Hygiene and Tropical Medicine, London WC1E 7HT, UK; 4 National Centre for Laboratory and Epidemiology, Vientiane, Laos; noikaseumsy@gmail.com; 5 Microbiology Laboratory, Khammouan Provincial Hospital, Thakhek, Laos; bacterialkm@gmail.com

**Keywords:** melioidosis, *Burkholderia pseudomallei*, Laos, Lao PDR

## Abstract

Melioidosis is clearly highly endemic in Laos, although the disease has only been diagnosed regularly in humans (1359 cases) since 1999, and only a single animal case has been microbiologically confirmed. *Burkholderia pseudomallei* is extensively and abundantly present in soil and surface water in central and southern Laos, but the true distribution of the disease across the country remains to be determined. Surveillance is almost non-existent and diagnostic microbiology services are not yet well established, whilst awareness of melioidosis is low amongst policy-makers, healthcare providers, and the public. It is hoped that this situation will improve over the next decade as the country rapidly develops, especially as this is likely to be accompanied by a further increase in the prevalence of diabetes, meaning that more people in this predominantly agricultural population will be at risk of contracting melioidosis.

## 1. History

The Lao People’s Democratic Republic (Laos) is a land-locked country in Southeast Asia with a population of approximately 6.5 million people, the majority of whom are subsistence farmers, although the country is developing rapidly. Melioidosis was not recognized in Laos until 1999 [1]. Given that Laos is adjacent to highly endemic parts of Thailand, it is likely that the bacterium that causes it has long been present in the environment but that it was not recognized due to a lack of laboratory facilities and awareness amongst healthcare staff. In fact, the detection of *Burkholderia pseudomallei* in the Lao environment preceded the detection of cases of human melioidosis, although this was not reported until later [2]. An even earlier case was almost certainly acquired in Laos during military action in 1954, although the diagnosis was made in a hospital in Vietnam [3].

## 2. Review of Melioidosis Cases and Presence of *B. pseudomallei*

### 2.1. Human

Diagnostic microbiology services for human infectious disease have been relatively underdeveloped in Laos compared withmost neighbouring countries such as Thailand, China, and Vietnam. The confirmation of melioidosis as an important public health problem in Laos followed the establishment of a research collaboration between the Microbiology Laboratory of Mahosot Hospital, one of the main central referral hospitals in the capital, Vientiane, and the Mahidol-Oxford-Research Unit in Thailand, which had been working on melioidosis in Ubon Ratchathani since 1986. This led to the creation of the Lao-Oxford-Mahosot Hospital-Wellcome Trust Research Unit (LOMWRU), which has supported the provision of diagnostic services for melioidosis, including the use of selective culture media and reagents (Ashdown’s agar and broth, latex agglutination and API 20NE) and collated data on culture-positive cases since 1999. Between 2000 and 2004, LOMWRU found *B. pseudomallei* in 14 patients with bacteraemia, representing 3% of positive blood cultures [4]. Since then, the number of cases diagnosed has increased each year, reflecting greater awareness of the disease amongst healthcare workers as well as the growing numbers of Lao people with underlying diseases that predispose to melioidosis such as diabetes, which was estimated to have a prevalence of 5.6% of the Lao population in 2016 [5].

In addition to receiving clinical samples from Mahosot Hospital itself, samples are sent to Mahosot Microbiology Laboratory from other hospitals in and around Vientiane and from other provinces, particularly associated with studies of the aetiology of fever in Luangnamtha, Salavan, and latterly Xiangkhouang provinces. In total, the LOMWRU/Mahosot Microbiology Laboratory has diagnosed 1232 cases of culture-positive melioidosis between 1999 and 2017. The majority came from Vientiane Capital and Vientiane Province, which represent the main catchment areas for Mahosot Hospital; however, since Mahosot Hospital acts as a central referral hospital for parts of Laos and accepts samples from other hospitals for the investigation of possible melioidosis, cases have been seen originating from every province in Laos except Luangnamtha. Details of these cases will be reported elsewhere.

In addition, support was provided by the Centre d’Infectiologie Christophe Mérieux du Laos (CICML) to establish a diagnostic microbiology laboratory in Khammouan Provincial Hospital in Thakhek in 2009, and staff were trained in the recognition of *B. pseudomallei* and invited to refer any isolates whose identity was uncertain to LOMWRU. Between 2010 and 2017, 79 patients were confirmed with melioidosis, all of whom came from Khammouan Province.

Between 2011 and 2016, the European Union provided €3,000,000 to support the further development of diagnostic capacity in Laos (https://ec.europa.eu/europeaid/case-studies/eu-support-epidemiology-and-laboratory-capacity-laos_en). Laboratory staff in Champasak, Savannakhet, Luangphrabang, Oudomxai, and Luangnamtha were trained in the identification of *B. pseudomallei* and asked to refer suspect isolates to the National Centre for Laboratory and Epidemiology (NCLE) in Vientiane for confirmation. Between 2012 and 2017, this resulted in the identification of a further 48 cases of culture-positive melioidosis.

**Figure 1:**
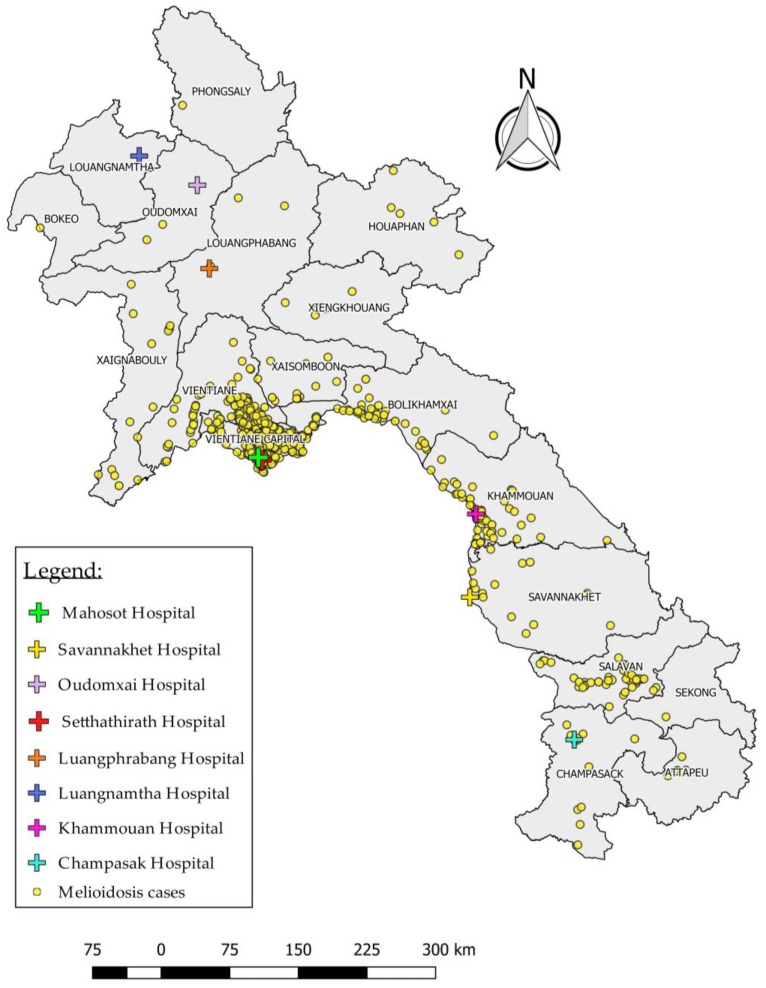
Location of homes of 1310 cases of melioidosis and hospital laboratories capable of making a diagnosis of melioidosis in Laos (data only available for 1310 of 1359 cases).

The data from these three sources have been combined in Table 1, and Figure 1 presents a map showing the distribution of cases and laboratories capable of confirming the diagnosis. An additional 19 Lao patients diagnosed in Thailand [6] and two additional travel-associated cases [7,8] have not been included in the table. Undoubtedly, these figures only represent the ‘tip of the iceberg’ in Laos, and the distribution of cases across the country is subject to considerable sampling bias. There are still large areas of the country that do not have access to good diagnostic laboratories. Awareness of melioidosis amongst both physicians and laboratory staff remains low, and, even where laboratories do exist, the use of diagnostic microbiology has not yet been fully assimilated into routine clinical practice. The fact that patients usually have to pay for diagnostic tests acts as a further disincentive to investigating the aetiology of infection.

### 2.2. Animal

Animal meliodosis is undoubtedly present in Laos, although to date only a single case, in a goat, has been confirmed by the LOMWRU laboratory (Newton, P.N.; Vongsouvath, M. (LOMWRU, Vientiane, Lao PDR). Unpublished observations, 2003). Animal surveillance in Laos is limited. Furtherstudies of melioidosis in animals are just beginning to be initiated.

### 2.3. Environment

Several studies have demonstrated that *B. pseudomallei* is widespread in the environment, at least in central and southern Laos, although the country has not been extensively and systematically sampled to determine the true nationwide distribution of the organism. *B. pseudomallei* was first isolated in 36% of 110 soil samples collected in and around Vientiane in 1998 [2]. A subsequent study found the organism to be abundantly present in soil in Salavan, but not in a transect from eastern Vientiane to Xiangkhouang [9]. A single isolate from soil in Luangnamtha was reported in this study, although subsequent studies of this isolate have shown that it was actually *Burkholderia cepacia* that cross-reacted in a *B. pseudomallei*-specific latex agglutination test (Dance, D.A.B. (LOMWRU, Vientiane, Lao PDR). Unpublished observations, 2011). *B. pseudomallei* was later confirmed to be present in both soil and surface water in Salavan, including the Sedone river [10]. However, these earlier studies were undertaken using culture alone, which may have underestimated the presence of *B. pseudomallei* [11]. *B. pseudomallei* was also found to be readily isolated from a rice paddy in Vientiane about 56 km from Vientiane’s capital [12]. More recently, a nationwide study of the presence of *B. pseudomallei* in the Mekong and its tributaries in Laos demonstrated its presence in 9% of the rivers in the dry season and in 57% of the rivers in the rainy season, mainly in turbid river water with associated *B. pseudomallei*-positive sediments, and exclusively in the south and centre of the country, suggesting that rivers may be useful in assessing the distribution and aquatic dispersal of *B. pseudomallei* [13].

## 3. Current Recommendations and Availability of Measures against Melioidosis

### 3.1. Surveillance Systems and Reporting

#### 3.1.1. Human

Melioidosis is not statutorily notifiable in Laos and there is no formal surveillance system for human melioidosis. Data are currently only collated by researchers with an interest in the disease as above. Informally, both LOMWRU and the Thakhek laboratories send monthly anonymized reports of episodes of bacteraemia (and other *B. pseudomallei* isolates in the case of the Thakhek laboratory) to the Ministry of Health and other relevant organisations.

#### 3.1.2. Animal

There is no surveillance system for animal melioidosis in Laos.

### 3.2. Guidelines

Melioidosis was included in a manual on diagnosis and treatment of infectious diseases produced (in Lao/English and Lao/French editions) by the Institut de la Francophonie pour la Médecine Tropicale du Laos in 2002 (updated in 2004) that was approved by the Minister of Health. Melioidosis was also included in the ‘National Treatment Guideline’ (fourth edition) in Lao that was issued in 2012. Both of these were distributed to district and provincial hospitals with the aim of improving the quality of management of infections, including melioidosis, but it is not known how widely available they are and whether they are followed. Neither is available online or outside Laos. The recommendations are also not entirely consistent with current international consensus guidelines on melioidosis treatment [14]. In addition, advice about clinical and epidemiological clues to the diagnosis, which samples to submit, and how to treat patients, have been distributed to users of the Mahosot Microbiology Laboratory through the Mahosot Microbiology Review, an internally distributed newsletter which is available both in English and Lao languages. Diagnostic guidance is contained in a laboratory user manual for the Mahosot laboratory, which is to be issued shortly, and plans are in place to include revised treatment guidelines in updated national antibiotic guidelines that are under development.

## 4. Awareness of Melioidosis

Awareness of the disease amongst health professionals has gradually increased in the area in and around Vientiane since the establishment of LOMWRU but is otherwise patchy, although some clinicians in provinces of relatively high incidence (e.g., Salavan and Savannakhet) regularly consider the diagnosis and manage patients accordingly.The disease is not yet considered a priority by the Ministry of Public Health. Although no formal surveys have been conducted, awareness amongst the public is likely to be even lower than that in Thailand [15].

## 5. Major Achievements

Activities relating to melioidosis in Laos are at a very early stage. A case series of the first 1088 patients with melioidosis is in preparation and it is planned that a national workshop, analogous to those that have taken place in neighbouring countries such as Cambodia, will be held in the near future. It is hoped that this will lead to the establishment of a national network of clinicians and researchers with an interest in the disease.

## 6. Current and Future Challenges

Based on the experience at Mahosot Hospital, it is likely that there are still hundreds of people dying of undiagnosed melioidosis in Laos each year, and this is likely to increase as diabetes becomes more common, as it has in neighbouring countries [16]. The biggest challenges facing those who wish to reduce this burden are the relative under-development of diagnostic microbiology services across the country, the lack of a ‘culture of culture’ amongst clinicians, and a lack of awareness of the disease amongst policy-makers, healthcare providers, and the public. It is hoped that significant progress can be made in these areas over the next decade.

## Figures and Tables

**Table 1:** Cases of culture-positive melioidosis diagnosed in Laos ^1,2^.

Province	1999	2000	2001	2002	2003	2004	2005	2006	2007	2008	2009	2010	2011	2012	2013	2014	2015	2016	2017	TOTAL
Attapeu									1	1		1			2	3			1	9
Bokeo												1								1
Bolikhamxai ^1^					1	2	3	3	3	3	2	3	2	4	8	8	7	7	12	68
Champasak ^1^			1	1				1		2					1	4		3	1	14
Houaphan												1	1	1		1			1	5
Khammouan ^1^							1	3		2		10	12	19	12	10	6	8	17	100
Luangnamtha																				0
Luangphrabang																1	2			3
Oudomxai												1							1	2
Phongsali																1				1
Salavan										2	6	4	4	3	3	11	9	4	15	61
Savannakhet ^1^												1	1		1	3	6	1	2	15
Vientiane Province ^1^		1	1	1	1	7	7	9	15	11	19	33	10	13	28	32	33	30	36	287
Vientiane Capital ^1^		2	6	4	5	25	20	27	51	31	22	58	41	57	66	65	81	108	73	742
Xaignaburi					1			1	1	1		2		1		1		4	4	16
Xekong										1										1
Xaisomboun ^3^	1				1				1		1	2			2	2	3	2	2	17
Xiangkhouang ^1^												1			1	1		1	1	5
Unknown ^1^						1	2						1				2	5	1	12
TOTAL	1	3	8	6	9	35	33	44	72	54	50	118	72	98	124	144	149	173	167	1359

^1^ Pooled data from LOMWRU, Thakhek Hospital and NCLE. ^2^ Data reflect patient’s home village (where known) but not necessarily place of acquisition of melioidosis. ^3^ Province established in 2014.
